# An antimicrobial stewardship program initiative: a qualitative study on prescribing practices among hospital doctors

**DOI:** 10.1186/s13756-015-0065-4

**Published:** 2015-06-04

**Authors:** Brita Skodvin, Karina Aase, Esmita Charani, Alison Holmes, Ingrid Smith

**Affiliations:** Norwegian Advisory Unit on Antibiotic use in hospitals, Department of Research and Development, Haukeland University Hospital, 5021 Bergen, Norway; Department of Health Studies, University of Stavanger, 4036 Stavanger, Norway; NIHR Health Protection Research Unit in Healthcare Associated Infection and Antimicrobial Resistance, Imperial College London, Du Cane Road, London, W12 0NN UK

**Keywords:** Antimicrobial use, Prescription practices, Hospital doctors, Antimicrobial resistance, Antimicrobial guideline, Qualitative research

## Abstract

**Background:**

Norway has a low, but increasing prevalence of resistance and few antimicrobial stewardship initiatives. When developing stewardship interventions, an understanding of the determinants of antimicrobial prescribing is needed. We report on the first qualitative study investigating factors influencing doctors’ antimicrobial prescribing practices in Norwegian hospitals.

**Methods:**

Qualitative semi-structured interviews were conducted with 15 Norwegian hospital doctors prescribing antimicrobials to adult patients. Interviews were transcribed verbatim and thematic analysis was applied to analyse the data.

**Results:**

Colleagues, in particular infectious disease specialists, microbiology test results and the newly published national guideline on antimicrobials were identified as key factors influencing antimicrobial prescribing practices. Delayed availability was a barrier for the utilization of microbiology test results and increasing clinical experience overrides the influence of the national guideline.

Patient assessment, informal training by experienced colleagues, and infectious disease specialists replacing managers in promoting prudent prescribing policies, also influenced prescribing practices.

**Conclusion:**

This study identified the following contextual factors that need to be addressed when developing antimicrobial stewardship programs in Norway: a common work practice for seeking collegial advice, logistics of microbiology test results, and formal leadership and systematic training on prudence. Other countries initiating stewardship programmes may benefit from performing a similar mapping of facilitators and barriers, to identify important stakeholders and organisational obstacles, before developing sustainable and tailored antimicrobial stewardship interventions.

**Electronic supplementary material:**

The online version of this article (doi:10.1186/s13756-015-0065-4) contains supplementary material, which is available to authorized users.

## Background

Though several countries have antimicrobial stewardship programmes (ASPs) in place [1], many are initiating stewardship activities, including such diverse countries as India and Norway [2, 3]. In Norway antimicrobial resistance (AMR) rates are low, but increasing, and antimicrobial consumption, in particular broad spectrum antimicrobials, has increased the last 20 years [4, 5]. In 2013, overall sales of antimicrobials were 20.0 Defined Daily Doses (DDD) per inhabitant per day. Hospitals are responsible for around 7 % of the total antimicrobial consumption [4].

The increasing national and international threat of AMR has highlighted the need for interventions to contain the low rates of AMR in Norway [3]. In an upcoming Norwegian action plan for containment of AMR mandatory components of ASPs and audits assessing the quality of the prescriptions will be addressed, filling the present void. “The National Advisory Unit for Antibiotic Use in Hospitals” (KAS) is to coordinate these initiatives in Norwegian hospitals.

The Norwegian healthcare system operates predominantly through government led health services and hospitals. The country has a dispersed geography with many small -, some medium- and a few large university hospitals. Many of the smaller hospitals lack on-site microbiology laboratories and infectious disease specialists (ID-specialists). Furthermore, clinical microbiologists and -pharmacists are not established professions, leaving antimicrobial prescribing decisions to be made by doctors alone. In July 2013, a new national guideline on antimicrobials was published, replacing local guidelines [6]. The guideline was developed with contribution from over 80 hospital doctors, mainly ID- specialists and is only available online.

ASPs have proven to be efficient in the short term, with no clear evidence of what are the successful components for a sustainable change in prescribing practices [7, 8]. A systematic review of antimicrobial prescribing studies in hospitals suggests that sustainability of ASPs may be improved with a better understanding of behavioural determinants of prescribing [9]. Another review concludes that cultural, contextual and behavioural factors need to be addressed to influence antimicrobial use [10]. Both qualitative and quantitative studies have been performed on the topic [11–18], however, we report on the first qualitative study in Norway investigating factors influencing antimicrobial prescribing practices among hospital doctors.

## Methods

### Study design

An explorative qualitative design using a semi-structured interview methodology was chosen to investigate factors influencing antimicrobial prescribing practices among hospital doctors [19, 20]. Face to face interviews were preferred over focus groups to reduce bias of social pressures between informants’ positions and specialities, preventing them from expressing their opinions freely.

### Study interview guide development

An interview guide was developed based on a literature review and individual face to face conversations with six key informants (hospital doctors), purposively sampled from two hospitals in Western Norway [10, 13, 21, 22]. Open ended questions were used to conduct the conversations. The six dimensions (structural, political, cultural, educational, emotional and physical) of healthcare quality identified by Bate, Mendel and Robert were applied as a framework to analyse data from the conversations and structure the interview guide [23]. Analysis of the key informant conversations identified two additional dimensions to the guide (patient- and hospital characteristics) and informed the detailed questions of the interview guide (full interview guide in additional file 1).

### Recruitment of participants

Author IS (study project manager) requested the Directors of development and research in all health trusts in Norway via e-mail to invite individuals to participate in the study. Some disseminated the invitation by e-mail asking for volunteers, and others selected candidates from the list of employees. Only doctors prescribing antimicrobials to adult patients and working in hospital wards were qualified for inclusion, including ID-specialists.

Initially, 55 doctors were identified by the Directors of development and research as eligible to participate in the study. To secure a rich diversity, 22 of them were consecutively selected based on age, gender, specialty, clinical experience, hospital (local-, regional- or university hospital) and geography. Author BS informed the 22 doctors about the study and personally invited them to participate by e-mail. Seven doctors did not respond to the invitation. Saturation of empirical themes was reached after ten interviews, however, to fulfil the criteria of diversity, 15 doctors were interviewed [24, 25].

### Interviews

Interviews took place between October 2013 and January 2014. All interviews were recorded and transcribed verbatim. Author BS, an ID-specialist and PhD student trained in qualitative methods conducted and transcribed the interviews. They were performed at the participants’ work place within working hours. The participants were not informed about the interviewers’ background, but were told if they asked.

### Analysis

Thematic analysis was applied to the transcripts using a combined deductive and inductive approach [20, 26, 27]. Two researchers (BS, IS) read all the transcripts independently, and a third researcher (KA) read a major sample of them. The three researchers independently listed the emerging themes and through discussions agreed on preliminary themes. One researcher (BS) identified quotes in all the transcripts reflecting each theme and developed preliminary subthemes. Subsequently quotes reflecting each subtheme were categorized, and corresponding descriptions were developed. Themes, subthemes and descriptions were then discussed by the three researchers, leading to reorganising, renaming and elimination of some themes and subthemes. This procedure was then repeated for themes and subthemes requiring further analysis. Final conclusion on themes, subthemes and descriptions were conducted through discussions and agreements between all three researchers (Table 2).

### Ethics

The Regional Committee for Medical and Health Research Ethics considered the study to only need approval by the Data protection officer representing The Norwegian Data Protection Authority, from which it was approved (2013/6960). All interviewees signed an informed consent.

## Results

Fifteen doctors from 13 hospitals and five major medical fields (internal medicine, surgery, oncology, neurology and intensive care) were interviewed. Two of the interviewees were ID- specialists (Table 1). Duration of interviews ranged from 36 to 68 min (average 54 min).

**Table 1:** Demographics of participants

Variable numbers	
Male/female	7/8
Age 25–35 years	6
Age 36–45 years	5
Age 46–55 years	2
Age 56–65 years	2
Interns/residents/consultants	2/5/8
Internal medicine	4
ID-specialists	2
Surgery^a^	4
Other medical fields^b^	3
Health trusts represented	9/20
Local hospitals represented	6
Regional hospitals represented	5
University hospitals represented	4

^a^Orthopedic, gastrointestinal, urology, gynecology

^b^Oncology, neurology, intensive care

The participants describe an antimicrobial prescribing practice in Norwegian hospitals which mainly involve interns and residents (doctors training to become specialists). In smaller hospitals, interns are the only doctors present in the emergency departments, whereas in bigger hospitals they work alongside residents. Normally interns discuss patients with residents who, when lacking sufficient knowledge and experience, discuss the patients with consultants. Consultants receive updates on hospitalized patients on morning and afternoon handover meetings. Antimicrobial treatment initiated in patients hospitalized during daytime is evaluated on evening rounds by a resident or a consultant on call. At hospital wards, consultants in general play the role of supervisors and attend ward rounds at variable frequencies. ID-specialist services vary greatly between hospitals. Some have ID-specialist consultants on-site, who perform counselling by phone or bedside, and may do systematic ward rounds, for instance at intensive care units. Other hospitals lacking ID-consultants obtain advice by phone from hospitals possessing this expertise. Nationwide ID-specialists are available by phone day and night all year.

In the following, we will use the six main themes that emerged from the analysis to describe the key factors influencing hospital doctors’ practice when prescribing antimicrobials; colleagues, microbiology test results, national guideline, training, patient assessment and leadership (Table 2).

**Table 2:** Description of the identified themes

Quotes	Description	Subthemes	Themes
Concerning antibiotic treatment, we follow a simple algorithm, but when things get complicated, we collaborate with the ID-specialists, and intensive care doctors, of course. [Consultant, gastro surgery] (C1)	The ID-specialist is the primary collaborator when treating difficult infectious disease patients	ID-specialists	Colleagues
Working as a resident, I first phone the consultant on call. However, often you end up phoning the resident on call at the department of internal medicine. Occasionally they can give you some advice, or they consult their consultants. A couple of times I have called the ID-specialist at the University hospital. [Resident, oncology] (C2)	When the ID-specialist is not readily available several other colleagues contribute to the choice of AB-treatment; More experienced colleagues in the wards and on call, internists, especially pulmonary doctors and microbiologists can provide input on AB treatment	Other colleagues	
We put great effort into obtaining specimens, preferably several specimens, in order to be sure that we use an adequate antifungal and not just Fluconazole. Our experience is that we more frequently, more often use other drugs, but then again, in accordance with resistance data. [Consultant, intensive care] (M1)	Microbiology test results are considered an important contribution to the treatment; Great effort is put into obtaining cultures and to check up on the preliminary results in order to adjust treatment accordingly.	Priority	Microbiology test results
If it has not been transferred to the electronical medical record, it’s not there. But it’s there. They are just waiting for the final resistance data. In other words, the test results are there, but it takes two or three days before they show up on the screen. So maybe.., yes. No, people just need to know that they can make a phone call. [Resident, internal medicine] (M2)	Microbiology reports become available very late to the clinician. The clinician tries to solve this by phoning to the lab, and vice versa.	Availability	
Our systems do not let us check up on what tests have been obtained. You actually have to call and ask: “Have you received the specimen so and so?” Or else, you would have to wait for the results for another two to three days. Once it is available, it is shown in the electronical medical record in the section for laboratory results. [Consultant, ID-specialist] (M3)			
It’s perfectly okay as long as you use it, you’re safe. No one can hold anything against you as long as you treat according to the guideline. It really makes you feel safe when on call. [Intern, internal medicine] (N1)	When knowledge and experience are insufficient, the guideline is perceived as a useful and supportive tool. The guideline’s significance however decreases with increased experience and knowledge.	Experience	National guideline
Well, I try to stick to the guideline, most of the time. If I do not, I normally have good reasons not to. But, I do not always agree with it. And I try to justify it if I do not follow it. [Consultant, ID-specialist] (N2)			
The computer works incredibly slow here. It is very annoying when logging on, that is. You just sit there and twiddle you thumbs for… That’s when it would have been great to have an app, just great. [Intern, internal medicine] (N3)	Suboptimal IT-systems impairs the availability of the guidelines. Distribution on several platforms would promote the availability	Availability	
..we have checklists for items they have to check out. And the antibiotic guideline is one among them. That’s how we somehow tell them this is to be complied with, and also to be sure that they know how to find it. [Consultant, internal medicine] (N4)	The guideline is used to promote AB policy	Promoting policy	
Education mainly takes place at the end- of- shift meetings, that is. Much is embedded in each of the cases we discuss. [Consultant, orthopedics] (T1)	Training is mainly informal and unsystematic; Lectures are held irregularly. However, training comes mainly from discussing clinical cases and observing more experienced colleagues	Informal and unsystematic	Training
Discussing with ID-specialists, but also observing how other doctors on call treat patients and discussions at the end- of- shift meeting. [Resident, internal medicine] (T2)			
There is no scheduled training, no. You’re expected to possess that knowledge, which you don’t have as an intern, because, it’s too theoretical. To have a guideline, -it is presented to you early on.. Just check the guideline, just use it. And you end up reading about it yourself. [Resident, internal medicine] (T3)	The national guideline is used as a substitute for the formal training	Guideline	
Sometimes, in the emergency department when your findings are inconclusive, you broaden the initial therapy. They keep telling me: “Try not to use broad spectrum as much,” but once in a while you just have to, and it’s is okay to a certain degree. Patient first, so to speak. [Intern, internal medicine] (P1)	When patient history, findings and diagnostic tools are inconclusive it feels safer to prescribe antimicrobials, than not. For the same reason broad- spectrum therapy often is chosen	Inconclusive conditions	Patient assessment
It depends on clinical judgement, and the patient’s clinical condition. If he is in very bad condition, fulfilling all the sepsis criteria, and has an unstable blood pressure and everything, only the broadest spectrum. [Consultant, urology] (P2)	Severity of disease determines the intensity of treatment; Threshold for starting AB, prescribing broad spectrum agents and prolonging therapy is lowered	Severity of disease	
No, I’m not quite sure whether I can call it politically incorrect, but severely ill neutropenic patients are given Meropenem although it’s possible that they shouldn’t be given any antibiotics at all, but at the same time I think that… [Consultant, internal medicine] (P3)			
I may have become better at waiting. In most cases, you have much more time than you expect. And in that case, you can wait until you know some more. [Resident, internal medicine] (P4)	Clinical experience facilitates dealing with the challenging conditions and to adopt a restrictive approach in antimicrobial treatment	Clinical experience	
No, it’s not on the agenda, that’s my experience. My impression is that we are free to do as we like. But, it doesn’t mean that we can go crazy. I think it would have been pointed out if we were to give everyone everything. I think it would have been put on the agenda. [Consultant, ID-specialist] (L1)	AB policy is to a small extent on the agenda of the hospital leaders	Priority	Leadership
NN is the leader of the infectious disease department, and he is on every end-of-shift meetings and so on. And it’s very.. people always say: “We give this and that, and I’m not sure that the ID-specialists agree.” And they sit there, and give corrections, or say: “Yes, but we have to resort to that,” or.. [Resident, internal medicine] (L2)	The ID-specialists advocates prudent AB use in discussions about clinical cases, typically on morning sessions.	ID-specialists	

### Colleagues

In daily clinical work, more experienced doctors are frequently consulted regarding antimicrobial therapies. When local expertise is insufficient, an ID-specialist is the desired colleague to seek advice from, mainly by phone, exemplified by the following quote: “Concerning antibiotic treatment, we follow a simple algorithm, but when things get complicated, we collaborate with the ID-specialists, and intensive care doctors, of course” (C1). The ID-specialist can also exert influence during handover meetings, through discussions regarding antimicrobial treatment of hospitalized patients.

Other specialities can also be influential, including pulmonologists and nephrologists when treating patients with pneumonia or kidney failure. Microbiologists may impact antimicrobial prescription when clinicians phone them for test results and choice of antimicrobials is discussed. One interviewee described the involvement of different colleagues in antimicrobial prescribing as follows: “Working as a junior doctor, I first phone the consultant on call. However, often you end up phoning the resident on call at the department of internal medicine. Occasionally they can give you some advice, or they consult their consultants. A couple of times I have called the ID-specialist at the University hospital“(C2).

### Microbiology test results

Doctors actively use microbiology test results when selecting antimicrobial therapy. Firstly, they emphasize obtaining specimens before starting antimicrobial treatment (M1). Secondly, they put a great effort into checking up on results, in order to adjust treatment. Lack of availability and timeliness is perceived as a limiting factor since test results are first made available when resistance data are complete (M2). In hospitals without a microbiology laboratory there is also the delay of specimen transport and transfer of results into separate electronic systems, leading to prolonged broad spectrum antimicrobial treatment, and patients being discharged before results are available. Clinicians try to overcome these obstacles by phoning the laboratory for preliminary test results (M2, M3), and laboratories phone clinical departments about important results such as positive blood cultures. However, opening hours of the laboratories are usually limited from morning to afternoon, six to seven days a week.

### National guideline

The national guideline is considered a useful tool by interns and inexperienced residents (N1). One less experienced doctor refers to the time period from when the local guideline was outdated until the new national guideline was published as follows: “When I was told that the guideline was outdated I panicked. What am I going to do, what am I going to use now? Fortunately, the new ones were then published.”

More experienced residents use the guideline as a reference for checking dosages and treating uncommon infectious diseases, whereas consultants, including ID-specialists, consider the guideline as less significant and emphasize the need to adjust treatment to individual patients (N2). They consider the guideline as a tool and not a law, and may point out its weaknesses.

The availability of the guideline is limited due to suboptimal IT-systems. Computers may be slow and the guideline hard to find, which is time consuming. Some participants therefore expressed a desire to have a print out, a pocket guide or a smart phone application (N3). Some doctors describe that the guideline is used as a tool to promote antimicrobial policy. Informal leaders (ID- specialists), and to a lesser extent formal leaders (hospital managers), point to the guideline as a national and local standard for antimicrobial treatment. This is especially stressed to new employees *e.g.* interns and locums (N4).

### Training

Lectures and courses in antimicrobial use are held, though irregularly. However, input from more experienced clinical colleagues is the most valued type of training (T1). Inexperienced doctors empasize supervision by experienced doctors when on call in the emergency room, and experienced doctors highlight discussions with ID-specialists. Learning may also come from sheer observation of how more experienced colleagues prescribe antimicrobials (T2).

The national guideline is used as a substitute for formal training. Experienced doctors or managers may refer to it as a useful tool to the less experienced. One resident said: “There is no scheduled training, no. You’re expected to possess that knowledge, which you do not as an intern, because it’s too theoretical. To have a guideline, -it is presented to you early on… Just check the guideline, just use it. And you end up reading about it yourself” (T3).

### Patient assessment

The influence of patient assessment on antimicrobial prescribing becomes evident in several settings. Firstly, when patient history, findings and diagnostics are inconclusive, or when infection is difficult to distinguish from cancer or rheumatic disorders, it feels safer to prescribe antimicrobials than not, and broad spectrum therapy is often chosen to secure adequate coverage (P1). Secondly, severely ill patients suffering from sepsis or significant comorbidities are often treated more aggressively with regard to initiation, spectrum, de-escalation and duration of antimicrobial therapy (P2, P3). Clinical experience facilitates dealing with these patients. According to the interviewees, experience makes it easier to identify the severely ill patients and to prescribe antimicrobials prudently (P4). The confidence to rely on narrow spectrum antimicrobials as adequate treatment for several severe conditions is only acquired with experience.

### Leadership

Hospital managers are not perceived as promoting antimicrobial policies. An ID-specialist said: “No, it’s not on the agenda, not that I know. My impression is that we are free to do as we like, but that doesn’t mean that we can “go crazy”. I think it would have been pointed out if we were to give everyone everything. Then it would have been put on the agenda” (L1). However, ID-specialists fill the void of managers and advocate prudence by promoting the guideline and the use of narrow spectrum antimicrobials, typically on handover meetings while discussing clinical cases (L2).

## Discussion

When exploring factors influencing hospital doctors’ antimicrobial prescribing practices the main themes identified were microbiology test results, colleagues and the antimicrobial guideline. Some of these results differ from what has been found in previous studies [11, 13, 28], and some have implications for the successful implementation of an ASP.

The most interesting finding was the participants’ emphasis on microbiology test results when prescribing antimicrobials and their frustration over delayed results. This has to our knowledge not been highlighted in previous studies. Experienced hospital doctors in Germany viewed microbiologists and laboratories as helpful in navigating antimicrobial treatment, but delayed results were not mentioned as a challenge [29]. A reason why delay came up as a major issue in our study may be the dispersed geography in Norway. Transferrals of specimens between hospitals and results back to the clinicians pose a major logistical challenge. Action to improve the line of communication between the laboratories and the clinics, both electronically and orally, is required to enhance support of clinical antimicrobial decision making. Future research should explore how leaders and staff at microbiology laboratories perceive the interaction with clinicians, as a basis for possible interventions on these lines of communication. Furthermore, studies show that antimicrobial stewardship teams can decrease time to appropriate therapy by close follow up of microbiological test results [30, 31], so establishing such teams in Norwegian hospitals is highly relevant.

Another major finding was the influence of colleagues on antimicrobial prescribing practice. Two studies conducted in Ireland and UK found a hierarchical system where senior colleagues had significant influence on prescribing practices of the doctors [28, 15]. Another study from the UK report on a prescribing etiquette where clinical leaders and senior doctors overrule the ID-specialists’ advice on antimicrobials [11]. On the contrary, our interviewees spoke of several colleagues as legitimate advisers, the ID-specialist being regarded as the superior. In accordance with our findings, a Swedish study found that all categories of doctors perceived the ID-specialists as important for antimicrobial prescribing and –resistance [12], and may express what is described as egalitarian Scandinavian work systems with a corresponding low consumption of antimicrobials [10]. Since our interviewees are responsive to advice from ID-specialists and ID-specialists are found to improve appropriateness of antimicrobial prescribing, they should be included in multidisciplinary antimicrobial stewardship teams [32]. However, many Norwegian hospitals lack ID-specialists, as well as clinical pharmacists and microbiologists, *i.e.* the traditional participants of ASP teams [33]. As a consequence, ASP teams may have to be staffed differently in the Norwegian model. Studies have shown that antimicrobial stewardship initiatives can be developed without the traditional staffing, structures and resources [34, 35]. The integration of nurses and other medical specialties should therefore be further explored in Norway.

A third major finding was that the doctors’ attitudes towards the national guideline correspond with level of clinical experience. Whereas interns and inexperienced residents are dependent on the guideline, senior doctors are more sceptical to it, which is in accordance with other studies [36, 37]. One interviewee, an ID-specialist, reported that he did not adhere to the guideline even though he had participated in developing it. This lack of adherence among senior doctors may be due to clinical autonomy and experience [11, 38]. In Norway it may also be explained by a gap in exposure to ASP interventions. Being on the brink of initiating nationwide ASP programmes, tailored audit and feedback to experienced doctors on prescribing and application of the guideline, may favourably be integrated in the programmes.

Furthermore, participants in the study report that their managers do not promote prudent use of antimicrobials. In hospitals with ID-specialists they may take the place of managers and promote prudence. However, when implementing an ASP, a formal leadership is considered essential to maintain the program [33]. Providing knowledge on AMR to raise awareness supplemented with local surveillance reports on antimicrobial use and -resistance, may be a useful strategy to engage with Norwegian hospital managers [39]. Another way to promote prudent antimicrobial prescribing practice could be to introduce formal and systematic training programmes [40, 41], especially for interns. Improved availability of the guideline is crucial and work is under way to provide access to the guideline in pocket guide and smart phone application formats.

The study has a few limitations. As interviewees were recruited by the Directors of development and research there may be a bias towards candidates with a special interest in antimicrobials.

Furthermore, the role of author BS (conducting interviews), being an ID-specialist, may affect the response from the participants and the interpretation of the results. However, this was tentatively handled by writing down preconceptions before conducting the interviews and by involving three authors with different backgrounds in crucial steps of the data analysis.

The sample of 15 interviewees met the methodological requirement of saturation of themes and diversity [24]. The sample addresses a wide range of constituencies as hospital size, age and professional background, securing diversity. ID-specialists’ prescribing practices differs significantly from other doctors’. We considered it important to include them in order to obtain a comprehensive picture of the antimicrobial prescribing practices in Norwegian hospitals.

## Conclusion

Our study has identified several contextual factors that influence antimicrobial prescribing in Norway, many which differ from those reported from other countries. These factors, such as a common work practice for seeking collegial advice, logistics of microbiology test results, and formal leadership and systematic training on prudence, need to be addressed when developing ASPs. This demonstrates the value of conducting a qualitative mapping of contextual factors before establishing antimicrobial stewardship initiatives. Other countries planning to implement ASPs may benefit from a similar mapping of facilitators and barriers, to identify important stakeholders and organisational obstacles, before developing sustainable and tailored ASP interventions.

## Additional file

Additional file 1:
**Interview guide on doctors’ prescribing of antimicrobials.**
