# Supplementary material for: An antimicrobial stewardship program initiative: a qualitative study on prescribing practices among hospital doctors
Source: Antimicrob Resist Infect Control. 2015 Jun 4;4:24. doi: 10.1186/s13756-015-0065-4 (PMC4465159; doi:10.1186/s13756-015-0065-4)
Supplement: Additional file 1: — Interview guide on doctors’ prescribing of antimicrobials. [file 13756_2015_65_MOESM1_ESM.docx]

Interview guide on

doctors` prescribing of antimicrobials

As you probably know, the aim of this project is to study hospital doctors` prescribing of antimicrobials. We want to explore what influences doctors when prescribing antimicrobials, in order to find measures that could help them when prescribing.

- Have you had time to read the information letter?

Then you are aware that this interview will be tape recorded, transcribed verbatim, and then analysed by a team of scientists. The recordings will be deleted within the end of 2014. We guarantee you confidentiality and you can withdraw from the study at any time until it has been published.

- Do you have any questions to what I have just said?
- Could you please sign the consent form?

Thank you so much for participating.

- Can you please tell me how you were recruited to the project?
- Can you tell me about your background? What I mean by that is your age, speciality, working position, responsibilities, which hospital(s) you have been working at and which university you attended.

**Culture**

Do you remember last you were on call and a patient was hospitalized with “an infection”?

- Could you please describe how you go about treating the patient? What did you do?
- How did you apply diagnostic tools?

Let`s say the patient suffered from an infectious disease originating from…

- How did you go about choosing antimicrobial treatment?

After two or three days you meet the same patient on ward, and she is still on the treatment that you prescribed.

- What do you have to bear in mind at this point?
- What would make you change the antimicrobial treatment?
- In what way does CRP influence your next move?
- What role do microbiological test results play at this point?
- How is it to find relevant information in the medical records on considerations concerning prescribed treatment?

When it comes to prescribing of antimicrobials;

- Could you please tell me, when do you find antimicrobial prescribing difficult?
- What do you do when that happens?
- What role do other doctors both from your department, but also from other departments, play for your prescribing of antimicrobials?
- And –nurses, what role do they play with regard to antimicrobial prescribing?

**Patient characteristics**

- Do you think that the patient and the patient´s condition influences your prescribing, and if so, in what way?

**Emotions**

- Could you please describe a situation where there was a discrepancy between the antimicrobial you should have prescribed and the one you actually prescribed?
- Could you explain how that happened?

**Education/knowledge**

- How is the training in antimicrobial prescribing at your department?
- What emphasis is there on antimicrobials in your educational program?
- What or who have taught you what you know about antimicrobials?
- How has increasing clinical experience altered your prescribing?
- What do you think of antimicrobial guidelines?

**Technology**

- What role do electronic tools play when you are prescribing antimicrobials? For instance, do you use the web, apps and the like?
- Are there any electronic solutions you miss when prescribing antimicrobials?

**Structure**

- Is there anything about the way your hospital is organized that could facilitate the prescribing of antimicrobials?

With organization, I mean schedules, time, staff, medical records, charts and so on.

- How is your access to information and help when you need it?
- How is the process of retrieving microbiological test results?
- What do you find beneficial with how this hospital or your department is organized, with regards to the prescribing of antimicrobials?

**Politics**

- Is your leader concerned about the use of antimicrobials, and if so, in what way?
- What are your thoughts on antimicrobial resistance?

**Characteristics of hospitals**

- Could you please tell me how prescribing of antimicrobials was done at any other hospital you`ve worked at?
- Why do you think prescribing practices differ between the hospitals?

**Final question**

Finally; having all the resources in world, if you were to give us some advice

- What measures do you think would be most useful in order to improve antimicrobial prescribing?
- Is there anything else you would like to add before we finish?

Thank you so much for your time!
